# New Insights in Cysticercosis Transmission

**DOI:** 10.1371/journal.pntd.0003247

**Published:** 2014-10-16

**Authors:** Carmen S. Arriola, Armando E. Gonzalez, Luis A. Gomez-Puerta, Maria T. Lopez-Urbina, Hector H. Garcia, Robert H. Gilman

**Affiliations:** 1 San Marcos Veterinary School, San Marcos Major National University, Lima, Peru; 2 Johns Hopkins University, Bloomberg School of Public Health, Baltimore, United States of America; 3 Universidad Peruana Cayetano Heredia, Lima, Peru; Universidad Nacional Autónoma de México, Mexico

## Abstract

*Taenia solium* infection causes severe neurological disease in humans. Even though infection and exposure to swine cysticercosis is scattered throughout endemic villages, location of the tapeworm only explains some of the nearby infections and is not related to location of seropositive pigs. Other players might be involved in cysticercosis transmission. In this study we hypothesize that pigs that carry nematodes specific to dung beetles are associated with cysticercosis infection and/or exposure. We carried out a cross-sectional study of six villages in an endemic region in northern Peru. We euthanized all pigs (326) in the villages and performed necropsies to diagnose cysticercosis. For each pig, we counted cysticerci; measured anti-cysticercus antibodies; identified intestinal nematodes; tabulated distance to nearest human tapeworm infection; and recorded age, sex, productive stage, and geographic reference. For the purpose of this paper, we defined cysticercosis infection as the presence of at least one cysticercus in pig muscles, and cysticercosis exposure as seropositivity to anti-cysticercus antibodies with the presence of 0–5 cysticerci. Compared to pigs without nematode infections, those pigs infected with the nematode *Ascarops strongylina* were significantly associated with the presence of cysticerci (OR: 4.30, 95%CI: 1.83–10.09). Similarly, pigs infected with the nematode *Physocephalus sexalatus* were more likely to have cysticercosis exposure (OR: 2.21, 95%CI: 1.50–3.28). In conclusion, our results suggest that there appears to be a strong positive association between the presence of nematodes and both cysticercosis infection and exposure in pigs. The role of dung beetles in cysticercosis dynamics should be further investigated.

## Author Summary

In endemic areas, pigs acquire cysticercosis when ingesting *Taenia solium* eggs that have been released into the environment in the feces of a person infected with *T. solium*. The present study has found evidence that players, such as dung beetles, might be involved in further dissemination of the parasite into the environment. Specifically, we found an association between helminths, for whom dung beetles act as an intermediate host, and porcine cysticercosis infection and exposure after adjusting for other porcine cysticercosis predictors such as distance to tapeworm carrier and age. Although the study does not evaluate dung beetles directly, parasites specific to dung beetles serve as a novel proxy to evidence the potential role of dung beetles in the epidemiology of cysticercosis. Therefore, it is important that further studies elucidate the role of other players in cysticercosis transmission in order to better explain the reemergence and persistence of cysticercosis after elimination and control efforts. In addition, vector populations could potentially be used as markers for cysticercosis in the communities.

## Introduction

Cysticercosis affects humans following the ingestion of *Taenia solium* eggs, generally by fecal-oral contamination. Once ingested, *T. solium* eggs turn into cysticerci. In humans, cysticerci establish primarily in the central nervous system and is the main cause of epilepsy in adults in endemic areas [1]. In Peru, the prevalence of human cysticercosis is relatively high in endemic areas [2]. The *T. solium* life cycle requires an intermediate host, the pig, to develop its larvae stage. When humans eat pork contaminated with cysticerci (*T. solium* larvae), *T. solium* tapeworms develop in their guts and then release eggs via human defecation. *T. solium* eggs turn into cysticerci when ingested by pigs. The presence of cysticerci in pigs can be observed macroscopically in the muscles as cysticerci are as large as rice grains. When cysticerci are viable, they are able to transmit the disease; cysticerci that are not able to transmit the disease are considered degenerated or non-viable cysts [3], [4].

Distance to human tapeworm carrier has been associated with swine cysticercosis infection [5], [6]. However, not all pigs around the tapeworm carrier become infected; in addition, some pigs far from the tapeworm carrier become infected or show seroprevalence to cysticercosis [6]. These findings suggest that an environmental vector may play a role in *T. solium* egg dispersion. We add evidence that the dung beetle may be such an environmental vector [7].

Pigs carry nematodes that require dung beetles as intermediate hosts to complete their life cycle [8], [9]. Because dung beetles feed exclusively from feces and have been previously described to have a role in disease transmission [8], [10] and because their role in *T. solium* transmission is uncertain, we tested the hypothesis that pigs that carry nematodes specific to dung beetles are associated with cysticercosis infection and/or exposure (Figure S1). For the purpose of this paper, we defined cysticercosis infection as the presence of at least one cysticercus in pig muscles, and cysticercosis exposure as seropositivity to anti-cysticercus antibodies with the presence of 0–5 cysticerci.

## Methods

### Ethics statement

This study was reviewed and approved by the Ethical Committee of Animal Welfare of the School of Veterinary Medicine, San Marcos Major National University, Lima-Peru (Authorization Number 2004-007) which adheres to the guidelines of the Council for International Organizations of Medical Sciences (World Health Organization) and the guidelines of the Office of Laboratory Animal Welfare (National Institutes of Health, USA).

### Study design

A cross-sectional study of six villages of an endemic region in northern Peru was carried out, and all pigs (326) in the villages were euthanized and necropsies performed to diagnose cysticercosis, as described by Lescano et al. (2007) [6]. Data on *T. solium* infection were collected: infection/non-infection, number of viable cysticerci in muscles, and number of degenerated cysticerci in muscle. Also, blood samples were obtained and seroprevalence for cysticercosis was determined by western blot test [11], [12]. In addition, data on other parasites observed at necropsy were gathered; parasites were further identified by genus and species. Other collected variables related to the pigs included age, sex, productive stage, village, household, and geographic reference (latitude, longitude).

### Statistical analysis

A new dichotomous variable for age was created for pigs less than and more or equal to nine months of age [13]. The logarithm of the distance (in meters) to the nearest tapeworm carrier was calculated for each pig [5]. In addition, another dichotomous variable was created for pigs with a positive western blot anti-cysticercus antibody test that have 0–5 cysts [14], as a measure of recent exposure to *T. solium* eggs [8], [11].

To study the association between cysticercosis infection and dung beetle nematodes, we first analyzed the bivariate associations between cysticercosis infection (viable cysticerci (PV), degenerated cysticerci (PD) and positive infection to any type of cysticerci (PIC)) and the nematodes *Ascarops strongylina* and *Physocephalus sexalatus*. In the same way, cysticercosis exposure (positive for exposure or seropositive (PE)) was also analyzed. Multivariable logistic regression (MLR) models were constructed to study the association between cysticercosis infection and exposure and *A. strongylina* and *P. sexalatus*, controlling for traditional risk factors for infection (distance to the nearest tapeworm carrier, sex, age). The four models were evaluated for the number of parameters (Akaike Information Criteria). The Huber/White Estimator was used to obtain robust standard errors to account for clustering. Data were analyzed using statistical software Stata/IC 10.0 (College Station, TX, US). P values≤0.05 were considered statistically significant.

## Results

### Descriptive statistics

The overall prevalence of cysticercosis in the six villages was 12.27% (40/326). In addition, the prevalence of viable and degenerated cysticerci was 5.52% (18/326) and 9.51% (31/326), respectively; and seroprevalence of pigs with 0–5 cysticerci was 52.45% (171/326). The pig population characteristics by villas are depicted in Table S1. Distance to the nearest tapeworm carrier ranged from 0 to 10,844 meters. Among all pigs, 54.91% were females and 45.09% were males. Also, the age of pigs ranged from 1 to 48 months.

Two nematode species were found during the pig necropsies: *A. strongylina* (Family: *Spirocercidae*, Order: *Spirurida*) and *P. sexalatus* (Family: *Physalopteridae*, Order: *Spirurida*) [9]. *A. stronglyina* and *P. sexalatus* were present in 17.79% (58/326) and 29.45% (96/326) of pigs, respectively (Table S1).

Based on analysis of age, we observed that the cysticercosis-infected pigs tended to be older than 10 months and closer to the nearest tapeworm carrier.

In addition, there were no substantial sociodemographic and agricultural differences between villages, which supported excluding village of our subsequent logistic regression models (Table S2). However, we used the robust estimate of the standard error to account for any clustering effect of the villages.

### Statistical models

The four MLR models are for PV (positive infection with viable cysticerci), PD (positive infection with degenerated cysticerci), PIC (positive infection with any type of cysticerci), and PE (positive exposure). The model with seven parameters was selected based on an Akaike information criterion (AIC) evaluation (AIC<10, Table S7) and scientific input [15]. The Hosmer-Lemeshow goodness-of-fit test confirmed a good fit of the data for the four models (p-value>0.05).

### Model interpretations

The odds of having viable cysticerci was 3.9 times higher in those pigs that carried *A. strongylina* compared to those pigs that did not carry *A. strongylina* after adjusting for sex, age, distance to the nearest tapeworm carrier, presence of *P. sexalatus* (p-value = 0.083, 95% CI: 0.83–18.6; Table S3). Likewise, the odds of having degenerated cysticerci was 3.1 times higher in those pigs that carried *A. strongylina* compared to those pigs that did not carry *A. strongylina* after adjusting for sex, age, distance to the nearest tapeworm carrier, and presence of *P. sexalatus* (p-value = 0.037, 95% CI: 1.07–9.31; Table S4). Moreover, the odds of having any type of cysticerci was 4.3 times higher in those pigs that carried *A. strongylina* compared to those pigs that did not carry *A. strongylina* after adjusting for sex, age, distance to the nearest tapeworm carrier, and the presence of *P. sexalatus*; this association was statistically significant (p-value = 0.001, 95% CI: 1.83–10.09; Table S5). Furthermore, the presence of *P. sexalatus* was positively associated with pigs positive to western blot that have 0–5 cysticerci (OR: 2.21, p-value = <0.001, 95%CI: 1.50–3.28; Table S6) adjusting for other risk factors (age, sex, distance to the nearest tapeworm carrier, presence of *A. strongylina*).

## Discussion

Our results show an association between the presence of nematodes and cysticercosis infection and exposure in pigs. Whereas *A. strongylina* was associated with cysticercosis infection, *P. sexalatus* was associated with cysticercosis exposure. The larvae of these two nematodes are transmitted by intermediate hosts; dung beetles [16], [17]. These results suggest that dung beetles may play a role in cysticercosis transmission dynamics.

To date, distance to the tapeworm carrier has been evaluated as the primary variable in explaining swine cysticercosis [6]. However, after adjusting for distance to nearest tapeworm carrier and other factors such as age and sex, we found a positive association between cysticercosis infection and the presence of *A. strongylina*. The presence of *A. strongylina* indicates that pigs have eaten dung beetles; therefore, dung beetles may play a role in swine cysticercosis infection. In addition, the other nematode species, *P. sexalatus*, was found to be associated with exposure to cysticercosis, suggesting that *P. sexalatus*' intermediate hosts may be playing a role in dissemination of low egg loads that provide sufficient exposure to cysticercus antigen and thereby conferring anti-cysticercus antibodies but no or low disease.

In a pilot study Gonzalez et al. 2007 (unpublished data) demonstrated the capacity of dung beetles to ingest *T. solium* eggs and reproduce the disease by orally infecting naïve pigs. Two western blot negative pigs were fed six dung beetles each in an experimental design. Pig #1 was fed dung beetles that were fed *T. solium* eggs three days prior. Pig #2 was fed dung beetles that were fed *T. solium* eggs three weeks prior. Each dung beetle harbored approximately 50 *T. solium* eggs. The two pigs were slaughtered after 60 days of infection. At necropsy, pig #1 had no cysticerci but showed a positive anti-cysticercus western blot, and pig #2 had 100 viable cysticerci, 6 degenerated cysticerci and was positive to anti-cysticercus western blot. Although limited by the number of pigs in this experiment, this pilot study shows that 1) dung beetles can ingest *T. solium* eggs and 2) pigs can become infected or antigen-exposed with cysticercosis when eating dung beetles that have ingested *T. solium* eggs. These findings reaffirm the observational evidence presented by Nichols and Gómez (2014) [18].

Although not assessed in this study, different dung beetle species may serve as intermediate hosts for different nematodes. A study in a cysticercosis endemic area in Peru showed that the most frequent dung beetle species were from the genera *Canthon* and *Deltochilum* [19]. These two species differentiate in that *Canthon* has affinity for human and bovine feces whereas *Deltochilum* has affinity for bovine and equine feces [19]. In addition, there might be differences in dung beetle ecology or characteristics that explain these different associations [20]. For instance, Verdú and Lobo (2008) observed different flying techniques in these two genera [21]. The present study indirectly assessed the potential role of dung beetles as paratenic hosts by analyzing data of nematodes that require dung beetles as an intermediate host. However, further studies may elucidate specific dung beetles species that are associated with cysticercosis transmission.

The importance of these findings lies in its implication for *T. solium* control and elimination programs in endemic areas. Elimination efforts have taken place in endemic areas in Peru [22]–[26], but these strategies have yet to eliminate the disease for more than two years [27]. Dung beetles may help explain the re-emergence of the parasite in controlled endemic areas [28], [29]. Dung beetles may also serve as potential markers for *T. solium*/cysticercosis in the community [30]. This study's finding of an association between dung beetle nematodes and swine cysticercosis infection and immune response encourages further investigation into the role that dung beetles play in cysticercosis transmission.

## Supporting Information

Figure S1 **Compartmental model for swine cysticercosis transmission dynamic.** Main predictors for pig infection or exposure to *T. solium* eggs are: 1) close proximity to the tapeworm carrier (human), and 2) consumption of potential *T. solium* egg carriers (dung beetles).
(DOCX)

Table S1 **Characteristics of the pig population, by villages.** Main characteristics of the pig population by villages (age, sex), and also prevalence of *cysticercosis*, *Ascarops strongylina*, and *Physocephalus sexalatus* determined by necropsy diagnosis.
(DOCX)

Table S2 **Characteristics of the villages.** General socio-demographic and socioeconomic characteristics of the villages.
(DOCX)

Table S3 **Multivariable analyses for the presence of one or more than one viable cysticerci (PV).** Results of descriptive and multivariable analysis for independent variables included in the model. For this table, the dependent variable is defined as the presence of one or more than one viable cysticerci (PV).
(DOCX)

Table S4 **Multivariable analyses for the presence of one or more than one degenerated cysticerci (PD).** Results of descriptive and multivariable analysis for independent variables included in the model. For this table, the dependent variable is defined as the presence of one or more than one degenerated cysticerci (PD).
(DOCX)

Table S5 **Multivariable analyses for the presence of one or more than one cysticerci either viable or degenerated or both (PIC).** Results of descriptive and multivariable analysis for independent variables included in the model. For this table, the dependent variable is defined as the presence of one or more than one cysticerci either viable or degenerated or both (PIC).
(DOCX)

Table S6 **Multivariable analyses for seroprevalence of pigs infected with 0–5 cysticerci (PE).** Results of descriptive and multivariable analysis for independent variables included in the model. For this table, the dependent variable is defined as seroprevalence of pigs infected with 0–5 cysticerci (PE).
(DOCX)

Table S7 **Akaike information (AIC) criterion differences for PV, PD, PIC and PE models.** Difference of Akaike information (AIC) criterion for model *i* relative to the minimum AIC among alternative models.
(DOCX)
