# Supplementary material for: New Insights in Cysticercosis Transmission
Source: PLoS Negl Trop Dis. 2014 Oct 16;8(10):e3247. doi: 10.1371/journal.pntd.0003247 (PMC4199528; doi:10.1371/journal.pntd.0003247)
Supplement: Figure S1 — Compartmental model for swine cysticercosis transmission dynamic. Main predictors for pig infection or exposure to T. solium eggs are: 1) close proximity to the tapeworm carrier (human), and 2) consumption of potential T. solium egg carriers (dung beetles). (DOCX) [file pntd.0003247.s001.docx]

Figure S1. Compartmental model for pig cysticercosis transmission.

Tapeworm in human (exposure)

Pig Infection /Exposure

Dung beetles

Distance(meters)
