# Supplementary material for: New Insights in Cysticercosis Transmission
Source: PLoS Negl Trop Dis. 2014 Oct 16;8(10):e3247. doi: 10.1371/journal.pntd.0003247 (PMC4199528; doi:10.1371/journal.pntd.0003247)
Supplement: Table S1 — Characteristics of the pig population, by villages. Main characteristics of the pig population by villages (age, sex), and also prevalence of cysticercosis, Ascarops strongylina, and Physocephalus sexalatus determined by necropsy diagnosis. (DOCX) [file pntd.0003247.s002.docx]

**Table S1. Characteristics of the pig population, by villages.**

|  | **Villa** | | | | | | Total (%) |
| --- | --- | --- | --- | --- | --- | --- | --- |
|  | Papayal | Chicama | El Cardo | Fernandez | Teniente Astete | Capitan Hoyle |  |
|  |  |  |  |  |  |  |  |
| **Swine population** |  |  |  |  |  |  |  |
| Total number (%) | 35 (10.74) | 26(7.98) | 30(9.20) | 26(7.98) | 73(22.39) | 136(41.72) | 326(100.00) |
|  |  |  |  |  |  |  |  |
| **Age** |  |  |  |  |  |  |  |
| <9 months | 26(74.29) | 19(73.08) | 12(40.00) | 18(69.23) | 38(52.05) | 63(46.32) | 176(53.99) |
| >= 9 months | 9(25.71) | 7(26.92) | 18(60.00) | 8(30.77) | 35(47.95) | 73(53.58) | 150(46.01) |
|  |  |  |  |  |  |  |  |
| **Sex** |  |  |  |  |  |  |  |
| Female | 17(48.57) | 14(53.85) | 22(73.33) | 16(61.54) | 39(53.42) | 71(52.21) | 179(54.91) |
| Male | 18(51.43) | 12(46.15) | 8(26.67) | 10(38.46) | 34(46.58) | 65(47.79) | 147(45.09) |
|  |  |  |  |  |  |  |  |
| **Prevalence of pigs with viable cysticerci** |  |  |  |  |  |  |  |
| Total number (%) | 0(0.00) | 1(3.85) | 8(26.67) | 2(7.69) | 0(0.00) | 7(5.15) | 18(5.52) |
|  |  |  |  |  |  |  |  |
| **Prevalence of pigs with degenerated cysticerci** |  |  |  |  |  |  |  |
| Total number (%) | 4(11.43) | 4(15.38) | 8(26.67) | 3(11.54) | 2(2.74) | 10(7.35) | 31(9.51) |
|  |  |  |  |  |  |  |  |
| **Prevalence of pigs with any cyst** |  |  |  |  |  |  |  |
| Total number (%) | 5(14.29) | 4(15.38) | 12(40.00) | 4(15.38) | 2(2.74) | 13(9.56) | 40(12.27) |
|  |  |  |  |  |  |  |  |
| **Seroprevalence of pigs infected with <5 cysticerci** |  |  |  |  |  |  |  |
| Total number (%) | 15(42.86) | 9(34.62) | 20 (66.67) | 11(42.31) | 41(56.16) | 75(55.15) | 171(52.45) |
|  |  |  |  |  |  |  |  |
| **Prevalence of *Ascarops strongylina* In pigs** |  |  |  |  |  |  |  |
| Total number (%) | 12(34.29) | 12(46.15) | 11(36.67) | 8(30.77) | 0(0.00) | 15(11.03) | **58(17.79)** |
|  |  |  |  |  |  |  |  |
| **Prevalence of *Physocephalus sexalatus* In pigs** |  |  |  |  |  |  |  |
| Total number (%) | 7(20.00) | 8(30.77) | 19(63.33) | 10(38.46) | 20(27.40) | 32(23.53) | **96(29.45)** |
