# Supplementary material for: New Insights in Cysticercosis Transmission
Source: PLoS Negl Trop Dis. 2014 Oct 16;8(10):e3247. doi: 10.1371/journal.pntd.0003247 (PMC4199528; doi:10.1371/journal.pntd.0003247)
Supplement: Table S2 — Characteristics of the villages. General socio-demographic and socioeconomic characteristics of the villages. (DOCX) [file pntd.0003247.s003.docx]

**Table S2. Characteristics of the villages.**

|  | **Villa** | | | | | | Total (%) |
| --- | --- | --- | --- | --- | --- | --- | --- |
|  | Papayal | Chicama | El Cardo | Fernandez | Teniente Astete | Capitan Hoyle |  |
|  |  |  |  |  |  |  |  |
| **Households** |  |  |  |  |  |  |  |
| Number of households | 13 | 11 | 11 | 41 | 9 | 30 | 115 |
|  |  |  |  |  |  |  |  |
| **Water sources** |  |  |  |  |  |  |  |
| Well | 13(100.0) | 4 (36.36) | 8 (72.73) | 38 (92.68) | 9 (100.00) | 29 (96.97) | 101 (87.83) |
| Creek | 0 (0.0) | 2 (18.18) | 2 (18.18) | 2 (4.88) | 0 (0.0) | 1 (3.33) | 7 (6.09) |
| other | 0 (0.0) | 5 (45.45) | 1 (9.09) | 1 (2.44) | 0 (0.0) | 0 (0.0) | 7 (6.09) |
|  |  |  |  |  |  |  |  |
| **Sanitary facilities(Latrine/Bathroom)** |  |  |  |  |  |  |  |
| Yes | 0 (0.0) | 2 (18.18) | 1 (9.09) | 28 (68.29) | 5 (55.56) | 9 (30.00) | 45 (39.13) |
| No | 13(100.0) | 9 (81.82) | 10(90.91) | 13 (31.71) | 4 (44.44) | 21 (70.00) | 70 (60.87) |
|  |  |  |  |  |  |  |  |
| **Rooms in the house** |  |  |  |  |  |  |  |
| <=4 | 11 (84.62) | 10 (90.91) | 9 (81.82) | 33(80.49) | 8(88.89) | 27(90.0) | 98 (85.22) |
| >4 | 2 (15.38) | 1 (9.09) | 2 (18.18) | 8 (19.51) | 1 (11.11) | 3 (10.0) | 17(14.78) |
|  |  |  |  |  |  |  |  |
| **Pig Farming** |  |  |  |  |  |  |  |
| No | 7(53.85) | 6(54.55) | 5(45.45) | 22(53.66) | 3(33.33) | 8(26.67) | 51(44.35) |
| yes | 6(46.15) | 5(45.45) | 6(54.55) | 19(46.34) | 6(66.67) | 22(73.33) | 64(55.65) |
|  |  |  |  |  |  |  |  |
| **Chicken Farming** |  |  |  |  |  |  |  |
| No | 2(15.38) | 1(9.09) | 3(27.27) | 5(12.20) | 3(33.33) | 6(20.0) | 20(17.39) |
| yes | 11(84.62) | 10(90.91) | 8(72.73) | 36(87.80) | 6(66.67) | 24(80.00) | 95(82.61) |
|  |  |  |  |  |  |  |  |
| **Cattle Farming** |  |  |  |  |  |  |  |
| No | 6(46.15) | 2(18.18) | 5(45.45) | 31(75.61) | 6(66.67) | 20(66.67) | 70(60.87) |
| **Yes** | 7(53.85) | 9(81.82) | 6(54.55) | 10(24.39) | 3(33.33) | 10(33.33) | 45(39.13) |
|  |  |  |  |  |  |  |  |
| **Goat Farming** |  |  |  |  |  |  |  |
| No | 0 (0.00) | 0(0.00) | 2(18.18) | 10(24.39) | 4(44.44) | 8(26.67) | 24(20.87) |
| Yes | 13(100.0) | 11(100.0) | 9(81.82) | 31(75.61) | 5(55.56) | 22(73.33) | 91(79.13) |
|  |  |  |  |  |  |  |  |
| **Number of inhabitants** | 53 | 46 | 44 | 171 | 42 | 118 | 474 |
|  |  |  |  |  |  |  |  |
| **Gender** |  |  |  |  |  |  |  |
| Female | 19(35.85) | 25(56.82) | 20(43.48) | 86(50.29) | 18(42.86) | 55(46.61) | 223(47.05) |
| Male | 34(64.15) | 19(43.18) | 26(56.52) | 85(49.71) | 24(57.14) | 63(53.39) | 251(52.95) |
|  |  |  |  |  |  |  |  |
| **Age** |  |  |  |  |  |  |  |
| Average age | 28.5 | 35.23 | 36.31 | 26.43 | 24.78 | 25.79 | 28.1 |
