# Supplementary material for: New Insights in Cysticercosis Transmission
Source: PLoS Negl Trop Dis. 2014 Oct 16;8(10):e3247. doi: 10.1371/journal.pntd.0003247 (PMC4199528; doi:10.1371/journal.pntd.0003247)
Supplement: Table S3 — Multivariable analyses for the presence of one or more than one viable cysticerci (PV). Results of descriptive and multivariable analysis for independent variables included in the model. For this table, the dependent variable is defined as the presence of one or more than one viable cysticerci (PV). (DOCX) [file pntd.0003247.s004.docx]

**Table S3. Multivariable analyses for the presence of one or more**

**than one viable cysticerci (PV).**

| Variables | Viable cysticerci | |  | Multiple Logistic Regression | | |  |
| --- | --- | --- | --- | --- | --- | --- | --- |
|  | n/N | % |  | OR | 95% CI | p-value | |
| Presence of *Ascarops strongylina* | 9/58 | 16 |  | 3.94 | 0.83-18.6 | 0.083 | |
| Presence of *Physocephalus sexalatus* | 9/96 | 9 |  | 0.25 | 0.02-2.72 | 0.258 | |
| Distance to tapeworm carrier | - | - |  | 0.45 | 0.35-0.57 | <0.001 | |
| Sex | 13/179 | 7 |  | 2.03 | 0.73-5.61 | 0.173 | |
| Age | 15/150 | 10 |  | 10.73 | 2.34-49.22 | 0.002 | |

Multiple Logistic Regression results were adjusted for presence of *Ascarops strongylina*, presence of

*Physocephalus sexalatus*, distance to nearest tapeworm carrier (logarithm of distance in meters),

sex (males as reference group) and age of pigs (< 9 months as reference group).
