# Supplementary material for: New Insights in Cysticercosis Transmission
Source: PLoS Negl Trop Dis. 2014 Oct 16;8(10):e3247. doi: 10.1371/journal.pntd.0003247 (PMC4199528; doi:10.1371/journal.pntd.0003247)
Supplement: Table S6 — Multivariable analyses for seroprevalence of pigs infected with 0–5 cysticerci (PE). Results of descriptive and multivariable analysis for independent variables included in the model. For this table, the dependent variable is defined as seroprevalence of pigs infected with 0–5 cysticerci (PE). (DOCX) [file pntd.0003247.s007.docx]

**Table S6. Multivariable analyses for seroprevalence of pigs infected**

**with 0-5 cysticerci (PE).**

| Variables | Seroprevalence for pigs <5 cysticerci | |  | Multiple Logistic Regression | | |
| --- | --- | --- | --- | --- | --- | --- |
|  | n/N | % |  | OR | 95% CI | p-value |
| Presence of *Ascarops strongylina* | 29/58 | 50 |  | 0.64 | 0.38-1.07 | 0.088 |
| Presence of *Physocephalus sexalatus* | 59/96 | 61 |  | 2.21 | 1.50-3.28 | <0.001 |
| Distance to tapeworm carrier | - | - |  | 0.98 | 0.85-1.12 | 0.729 |
| Sex | 98/179 | 55 |  | 1.26 | 0.80-2.01 | 0.328 |
| Age | 97/176 | 55 |  | 1.61 | 0.94-2.73 | 0.082 |

Multiple Logistic Regression results were adjusted for presence of *Ascarops strongylina*, presence of

*Physocephalus sexalatus*, distance to nearest tapeworm carrier (logarithm of distance in meters),

sex (males as reference group) and age of pigs (< 9 months as reference group).
