# Supplementary material for: New Insights in Cysticercosis Transmission
Source: PLoS Negl Trop Dis. 2014 Oct 16;8(10):e3247. doi: 10.1371/journal.pntd.0003247 (PMC4199528; doi:10.1371/journal.pntd.0003247)
Supplement: Table S7 — Akaike information (AIC) criterion differences for PV, PD, PIC and PE models. Difference of Akaike information (AIC) criterion for model i relative to the minimum AIC among alternative models. (DOCX) [file pntd.0003247.s008.docx]

**Table S7. Akaike information criterion (AIC) differences for PV, PD, PIC and PE models.**

| Alternative models |  | PV | PD | PIC | PE |  |
| --- | --- | --- | --- | --- | --- | --- |
|  | Number of parameters | Δi | Δi | Δi | Δi |  |
| E(y)=β_o_+β_1_*ascarops | 3 | 43.6 | 18.30 | 33.3 | 0 |  |
| E(y)=β_o_+β_1_*distance | 3 | 9.9 | 19.30 | 24.1 | 4 |  |
| E(y)=β_o_+β_1_*ascarops+β_2_*physocephalus | 4 | 45.4 | 20.30 | 34.9 | 0 |  |
| E(y)=β_o_+β_1_*ascarops+β_2_*distance | 4 | 8.2 | 14.20 | 13.4 | 1.8 |  |
| E(y)=β_o_+β_1_*ascarops+β_2_*physocephalus+β_3_*distance | 5 | 8.7 | 15.80 | 15.3 | 2 |  |
| E(y)=β_o_+β_1_*ascarops+β_2_*physocephalus+β_3_*distance+β_4_*sex | 6 | 9.2 | 15.20 | 13.2 | 3.5 |  |
| E(y)=β_o_+β_1_*ascarops+β_2_*physocephalus+β_3_*distance+β_4_*sex+β_5_*age | 7 | **0.9** | **0.80** | **8.5** | **1.7** |  |
| E(y)=β_o_+β_1_*ascarops+β_2_*physocephalus+β_3_*distance+β_4_*age | 6 | 0 | 0.00 | 0 | 0.7 |  |
| E(y)=β_o_+β_1_*ascarops+β_2_*distance+β_3_*sex+β_4_*age | 6 | 2.4 | 0.90 | 8.4 | 1.6 |  |
| Δi=AICi-AICmin; PV =positive infection with viable cysticerci, PD=positive infection with degenerated cysticerci, PIC=positive  infection with any type of cysticerci, and PE=positive exposure. | | | | | | |
